# Strengthening referral of sick children from the private health sector and its impact on referral uptake in Uganda: a cluster randomized controlled trial protocol

**DOI:** 10.1186/s12913-016-1885-5

**Published:** 2016-11-11

**Authors:** Esther Buregyeya, Elizeus Rutebemberwa, Philip LaRussa, Anthony Mbonye

**Affiliations:** 1 Makerere University College of Health Sciences, School of Public Health, Kampala, Uganda; 2 Department of Pediatrics, Colombia University, New York, USA; 3 Ministry of Health Uganda, Community Health Department, Kampala, Uganda

**Keywords:** Referral of under-five children, Febrile illness, Private sector, Higher level facility, Trial

## Abstract

**Background:**

Uganda’s under-five mortality is high, currently estimated at 66/1000 live births. Poor referral of sick children that seek care from the private sector is one of the contributory factors. The proposed intervention aims to improve referral and uptake of referral advice for children that seek care from private facilities (registered drug shops/private clinics).

**Methods/Design:**

A cluster randomized design will be applied to test the intervention in Mukono District, central Uganda. A sample of study clusters will implement the intervention. The intervention will consist of three components: i) raising awareness in the community: village health teams will discuss the importance of referral and encourage households to save money, ii) training and supervision of providers in the private sector to diagnose, treat and refer sick children, iii) regular meetings between the public and private providers (convened by the district health team) to discuss the referral system. Twenty clusters will be included in the study, randomized in the ratio of 1:1. A minimum of 319 sick children per cluster and the total number of sick children to be recruited from all clusters will be 8910; adjusting for a 10 % loss to follow up and possible withdrawal of private outlets.

**Discussion:**

The immediate sustainable impact will be appropriate treatment of sick children. The intervention is likely to impact on private sector practices since the scope of the services they provide will have expanded. The proposed study is also likely to have an impact on families as; i) they may appreciate the importance of timely referral on child illness management, ii) the cost savings related to reduced morbidity will be used by household to access other social services. The linkage between the private and public sectors will create a potential avenue for delivery of other public health interventions and improved working relations in the two sectors. Further, improved quality of services in the private sector will improve provider confidence and hopefully more clientelle to the private practices.

**Trial registration:**

NCT02450630 Registration date: May/9^th^/2015

## Background

About half of mortality in children under five years of age in sub-Saharan Africa is caused by malaria, pneumonia and diarrhea [1]. Almost 60 % of parents with febrile children in Uganda first seek care in the private sector, especially at drug shops [2], however they provide sub-standard care [3, 4]. A functioning referral system is a critical part of an appropriate health care delivery system [5]. Referral systems have been given particular emphasis in the integrated community case management (iCCM), which is the recommended strategy for management of childhood illness in Africa [6]. In many developing countries, referral is an essential part of preventing unnecessary deaths: primary health care workers should refer children with life threatening illnesses which they are unable to treat properly [7]. In Uganda, the Ministry of Health encourages referrals from dispensaries and health centres to district hospitals, from district hospitals to regional and the National Referral hospitals [8]. Uganda’s under-five mortality is high, currently estimated at 66/1000 live births [9]. Low referral rates of sick children is one of the contributory factors [7, 8], particularly in those who seek care from the private sector.

Previous research in Uganda and elsewhere in Africa has found that referral rates of sick children from lower levels to higher levels of care are extremely low [7, 10–13]. Several factors have been attributed to poor referral rates including; lack of money and high costs involved in referral, long distances to the referral health facilities, poor attitudes of health workers, lack of drugs at the referral health facilities, lack of involvement of fathers in the referral process and waiting for malaria drugs provided at the lower level of care to finish [7, 11, 13, 14]. We propose an intervention designed to strengthen referral from private providers to higher level facilities with the aim of improving child survival by getting sick children to an appropriate level of care in a timely manner. The study targets children aged less than five years, especially in rural areas who have poor access to health interventions. The proposed intervention aims to improve uptake of referral advice for children that seek care from private facilities (registered drug shops/private clinics). We describe the trial management structure, interventions, design, and analysis plan, as well as expected contributions that this study can make.

### Aim of the study

The aim of the study is to assess the effect of a strengthened referral system from the private sector on uptake of referral advice. The project addresses poor referral of children as one of the challenges in the reduction of under-five mortality in Uganda. The proposed intervention aims to improve uptake of referral advice for children who’s parents seek care from private facilities (registered drug shops/private clinics), by i) training VHTs to do community sensitization and raise awareness about saving money for referral at a household level, ii) training and supervision of providers in the private sector to diagnose, treat and refer sick children- training the private providers in iCCM and iii) regular meetings between the public and private providers (convened by the district health team) to discuss the referral system.

### Objectives

The protocol focuses on one primary and two secondary objectives:

Primary objective

1. To assess the effect of strengthening the referral system on uptake of referral of sick children who initially seek care in the private sector.

Secondary objectives

1. To explore factors which influence the referral or non-referral of sick children from the private sector.
2. To assess the effect of training private providers on appropriate case management for malaria, pneumonia and diarrhea.

## Methods

Referral is defined here as any child under 5 years of age who has been seen at a private practitioner and recommended for higher level care. The private sector providers participating in this project will include private clinics and registered drug shops.

### Setting

The study will be conducted in Mukono, central Uganda. The total population of the district is 583,600 and the majority, 88 % live in the rural areas. The district has an annual population growth rate of 2.3 % per annum and is inhabited by mainly the Baganda an indigenous ethnic group whose main occupation is subsistence agriculture [15]. The district was selected to implement the study because it is endemic for malaria. Mukono district is served by a network of health providers, including public, private not-for-profit and private for profit health care services, which makes the referral from private to public sector easy. The health policy in Uganda supports public-private partnerships to improve health outcomes [8].

### Design

This study is a cluster randomized trial with two arms. Twenty clusters in Mukono district, central Uganda will be assigned to a control or intervention in the ratio 1:1, Fig. 1. A cluster was defined as a parish or neighbouring parishes if the distance between any two private health facilities located in each of the parishes is <1 km (to minimize possible spill over). The intervention consists of three components; i) VHTs will be trained to do community sensitization and raise awareness about saving money at a household level for to pay for future referral if care is needed, ii) training of the private providers in iCCM and provide with them with subsidized drugs, i.e., Artemesinin Combination Therapy (Coartem®) for malaria, amoxicillin for pneumonia, RDTs, Zinc and Oral Rehydration Salts (ORS) to treat diarrhea among children aged less than 5 years. They will also be trained in the use rapid diagnostic tests (RDTs) and how to recognize and distinguish uncomplicated and severe malaria, diarrhea and pneumonia; supply unit-dose packaged Coartem® for children with uncomplicated malaria and then administer rectal artesunate prior to referring children with severe and complicated malaria to a higher level of care (i.e., a health centre II and above), Fig. 2. These private providers will be supervised on how they diagnose, treat and refer sick children, iii) regular meetings between the public and private providers (convened by the district health team) will be held to discuss the referral system. In the control arm there will be standard of care (no training of private providers in iCCM, no community sensitization and no meetings between the public and private providers).

**Fig. 1:**
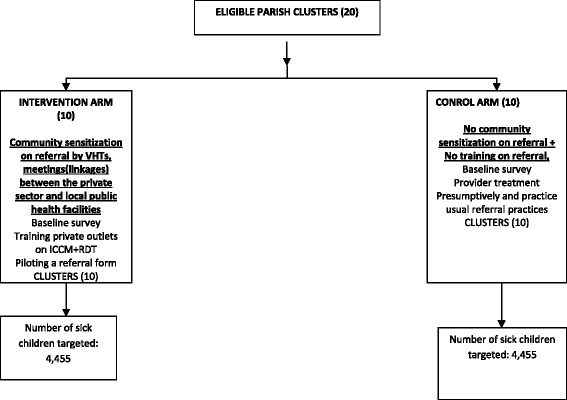
Profile of Study Design

**Fig. 2:**
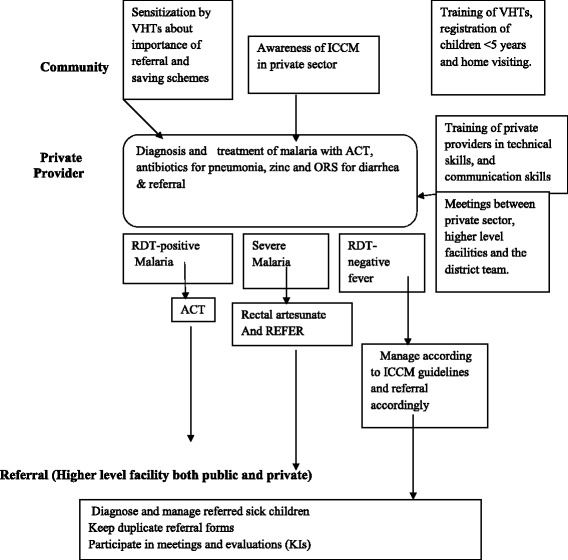
Summary Intervention Design

### Study hypothesis

Clusters (private providers) implementing a strengthened referral system from the private sector (through community sensitization, training of private providers in iCCM, supervision on how they diagnose, treat and refer sick children, regular meeting between the public and private providers on referral of sick children and provision of subsized drugs) will have more referral uptakes of sick children compared to those clusters that do not receive the study interventions.

### Principles for recruitment

#### Inclusion criteria

##### Cluster inclusion criteria

i) Contain more than 200 households to ensure a sufficient number of sick children visiting the private outlets
ii) Contained at least one registered drug shop/private clinic
iii) Contain a health centre II, the lowest public health facility where referral for early treatment is recommended.

##### Cluster exclusion criteria

i) Unregistered drug shop/private clinic
ii) Non-government health facility located within the same parish
iii) Fewer than 200 households in the parish where drug shop/private clinic is located
iv) Health centre II facility does not have a qualified health worker. Some government health HCIIs in Uganda are run by nursing aides.

##### Clinic/drug shop inclusion criteria

If they are registered and consent to participate in the study.

##### Clinic/drug shop exclusion criteria

If they have just been established within one year, because such newly established units may not have created a clientele of customers.

##### Patient inclusion criteria

i) Children aged <5 years with a fever, cough or diarrhea or had a history of any of the above when presenting at the private outlet or; ii) Axillary temperature ≥ 37.5 °C).

##### Patient exclusion criteria

i) Sick children with no fever, cough, diarrhoea or any symptom and ii) If they are visitors in the area and have not spent 2 weeks in the village.

### Randomization

Eligible clusters (20) will be randomly assigned as intervention or control arms. A process of restricted randomization will be utilised to balance cluster-level factors strongly associated with the primary outcome, or those expected to influence the effect of the intervention, between the study arms to ensure the credibility of the study and improve precision. The intervention is designed to address key barriers to effective treatment in the private sector; poor quality of care in the private sector, delay or no referral, and uptake of referral advice at a community level. Poor quality of care in the private sector may be attributed to inadequate training of providers in the private sector (in diagnosis and management of childhood illnesses); inadequate supervision and regulation; poor linkages and collaboration between the public and private sectors. Poor uptake of referral advice may be due to lack of awareness of severe signs for childhood illnesses and their consequences; and poor preparation of households in terms having finances for referral; and non-existent linkages between community structures and the public-private sectors. The intervention clusters will receive community awareness on referral, training of private providers in using RDTs/ICCM to treat and refer sick children, and supervision and regular meetings between the private and public sector, while the private providers in the control arm will only be trained in completing study tools i.e., filling in register and referral forms.

### Blinding

There will be no blinding put in place i.e., for the participants (private providers), investigators and data collectors. However, the person who will analyse the data will be blinded.

## Procedures

### Baseline survey

Prior to randomization, a baseline survey will be done to guide the randomization process. GPS coordinates for the private premises will be taken and these will be used to define the boundaries of a cluster. This is because we defined a cluster as a parish or group of neighbouring parishes if the distance between any two private outlets located in these parishes is <1 km. This was done to avoid contamination.

### Community sensitization

In the intervention arm, community consultation and sensitization will take place before implementing the intervention, to increase awareness about the need for referral within the community as summarized below;

- A meeting will be held with the district heath team to share the project objectives and focus on improving management of sick children in the district.
- Training of VHTs will take place in each of the study clusters. The village health teams will be mobilized at the village level before the start of recruitment, to inform communities within the study area of the trial, its purpose and what to expect.

### Training of private providers

Private providers in the intervention arm will be convened in a central place and trained in iCCM and using RDTs. This will be after they have consented to participate in the study. They will be trained in diagnosis, treatment and referral according to iCCM guidelines. The training will be carried out by experts in iCCM, together with the study team. In addition, the providers will be trained in filling registers and referral forms. They will be awarded training certificates that are to be displayed on their premises, as well as pictorial job aids that will be used to explain diagnosis, treatment and referral to sick children. Furthermore, they will be supplied with a placard identifying them as an approved provider of sick children with diagnostic tests.

Private providers in the control arm will be only trained in filling of registers and referral forms and not in iCCM. They will not be given certificates or job aids. All private providers in both arms will be trained to record data on sick children aged less than 5 years who seek care at private outlets for a period of 12 months/years after enrolment.

The following data will be captured in patient registers and treatment forms distributed in both the intervention and control clusters:

- Demographic (age, sex, relationship to caretaker, marital status of caretaker, education levels, rural/urban residence)
- Social-economic data (household income, ownership of household items that determine expenditure and consumption levels, address and telephone contacts of caretakers);
- Type of illnesses, treatment given, prescription of drugs, referral advice, children referred and where referred.

### Support supervision

Meetings between in-charges of health units and the private units in the intervention clusters will be convened at public health facilities on a quarterly basis to discuss the referral system. The VHTs and the private providers in the intervention arm will be supervised by the study team, the DHT and in-charges of health facilities. The supervision will be on a weekly basis in the first three months of the interventions and will be scaled down to monthly for the remaining nine months of the study period.

## Interventions

The barriers to referral from the private sector to higher level facilities will be addressed through an intervention with three components; i) VHTs to do community awareness on referral and initiate discussions on saving schemes for referral costs; ii) training and supervision of providers in the private sector to diagnose, treat and refer sick children, and iii) regular meetings between the public and private providers (convened by the district health team) to discuss the referral system.

- Intervention Arm – Community awareness on referral and training private providers in using RDTs/iCCM to treat and refer sick children and supervision and regular meetings between the private and public sector.

Training of the private providers in iCCM and provide them with subsidized drugs i.e., Coartem® for malaria, amoxicillin for pneumonia, RDTs, Zinc and Oral Rehydration Salts (ORS) to treat diarrhea among children aged less than 5 years. They were also be trained in the use of RDTs and how to recognize and distinguish uncomplicated and severe malaria, diarrhea and pneumonia; supply unit-dose packaged Coartem® for children with uncomplicated malaria and then administer rectal artesunate prior to referring these children with severe and complicated malaria to higher level facilities for care. Private providers will be given subsidized drugs. These private providers will be supervised on how they diagnose, treat and refer sick children.

## Training on referral

Health workers in the intervention arm will be trained on how to recognize and distinguish uncomplicated & severe malaria, supplying unit-dose packaged Coartem® to customers with uncomplicated malaria, and administration of rectal artesunate prior to referral for higher level treatment of sick children with severe and complicated malaria, diarrhoea and pneumonia. Private health facilities will be provided with low literacy visual aids (job aids) to help explain the symptoms, diagnosis, correct treatment of malaria and danger signs for severe disease. The private outlets will be expected to explain the pictorial instructions to customers, as well as give instructions on dosing and how to give the drug to the patient. The age and sex of sick children, onset of symptoms and promptness of treatment-seeking, diagnosis (including RDT result, where appropriate), tablets supplied and outcome (treatment and/or referral) will be routinely recorded by private outlets for all sick children seeking treatment for fever, cough or diarrhea.

All private outlets in the intervention arm will be trained to give pre-referral treatment using a rectal artesunate suppository to children with danger signs consistent with severe malaria [16] and to refer these cases immediately to the nearest health unit for further treatment. Private outlets will also be trained to refer any RDT-negative sick children with danger signs and/or a temperature >38.5 °C to the nearest health unit as an urgent case. This additional temperature criterion is based on the WHO definition of hyperthermia and is intended to help ensure that those with severe bacterial infections are seen at a health unit. The sick children will be referred using an emergency referral form, including details of symptoms and the use of pre-referral treatment.

For patients who are RDT negative with no serious symptoms, private providers will be trained to advise caretakers on tepid sponging, but to return for further assessment at the facility or a health facility should the symptoms persist or worsen.

In the control arm, private providers will be trained in completing study tools i.e., filling in referral forms and the rest will be business as usual (no training of private providers in iCCM, no community sensitization and no meetings between the public and private providers, Table 1.

**Table 1:** Comparison of the intervention and control arms

Intervention component	Intervention arm	Control arm
1. Training of private providers	Training in iCCM, RDTs and filling of patient registers and referral forms. Training will take five days.	Trained only in filling out patient registers and referral forms. Training will take one day.
2. Community sensitization by the VHTs	Yes	No
3. Supervision of the private providers by the district	Yes	No
4. Regular meetings between private and public sector to discuss referral	Yes	No

- Control Arm – Training of private providers in completing study tools i.e., filling in register and referral forms. No community awareness on referral

## Study outcomes and analytic methods

The primary analytic approach is intention to treat with children in the clusters in the intervention arm considered exposed to the intervention. Data for all of the primary and secondary outcome variables will be captured on the routine treatment recording forms and all consultations for fever, pneumonia and diarrhea routinely completed by drug shop vendors/private clinics. The outcome of referral will be recorded on the referral forms by health staff for referred sick children presenting to health facilities with a referral form. The primary trial outcome is the proportion of sick children referred from a private outlet that complete referral (seen at higher level facilities). The denominator includes all referrals of sick children with a history of fever, pneumonia and diarrhoea. Through review of records at the private health facilities, care takers whose children are referred in a specific period of time will be followed up in their respective communities to verify whether they took up referral. Secondary outcomes are; appropriate case management for malaria, pneumonia and diarrhea among children and factors which influence referral or non-referral of sick children from the private sector. Other outcomes include the proportion of sick children seeking care at private outlets within 24 h of onset of symptoms, and time between consultations at private outlets and uptake of referral at health facilities (referral facilities).

A cluster-level approach to the analysis will be used due to the small number of clusters per arm [17]. The proportion relating to this primary outcome will be calculated in each cluster. If the distribution of the proportions in each study arm is skewed a logarithmic transformation will be applied and the geometric mean proportions computed. To compare the intervention with the control arm point estimates based on these cluster summaries will be used to derive risk differences; using a weighted average of the cluster proportions, with weights proportional to the sample size for each cluster, since consultation rates differ between drug shops/private clinics and clusters. If the data have been log transformed, this will be the ratios of the geometric means. Corresponding 95 % confidence intervals will be computed assuming separate estimates of the variances in the two study arms, or if log transformed using an estimate of the pooled standard deviation of the log-transformed cluster summaries. The null hypothesis that there is no intervention effect will be tested using an unpaired *t*-test. Finally, an estimate of the coefficient of variation, *k*, will be provided. The overall mean proportion of sick children referred in the intervention arm, with 95 % confidence interval, will be computed using a random effects model to account for cluster sample. This analysis will be restricted to data from the treatment records of registered drug shop/private clinics enrolled in the intervention arm of the trial. A two-stage process will be carried out to adjust for covariates. At the first stage a regression model will be fitted incorporating the covariate(s) of interest, except the study arm. Expected values from this model will be compared with observed values by computing a residual, based on the difference between observed and expected, for each cluster. In the second stage, adjusted risk differences and 95 % CIs will be estimated as described above, replacing the cluster-level proportions with the covariate adjusted residuals. The covariates that will be adjusted for include balance criteria used in randomization at cluster level (primary endpoint at baseline); level of qualification, number of years working as a drug shop vendor/private clinic and/or health worker; age of sick child, education level of caretaker, and occupation of caretaker. A test for interaction will be carried out, and a 95 % confidence interval computed, using the methods of [18]. The above methods will also be used to analyse the other secondary outcomes; appropriate case management for malaria, pneumonia and diarrhea among children in the private sector and time between consultations at private outlets and uptake of referral at health facilities (referral facilities).

Factors which influence the referral or non-referral of sick children from the private sector will be explored through focus group discussions and key informant interviews. Coding of the transcripts will take place through an iterative process, with data being grouped into themes drawn from idea codes to generate a ‘node tree of ideas’.

Primary outcome:

- The proportion of sick children referred from the private sector that completes the referral process (seen at higher health facilities), Table 2.

**Table 2:** Period of evaluation and source of data for each endpoint

Endpoint	Source of data	Period of evaluation (start date – end date)
Primary endpoints:		
Uptake of referral advice	Referral forms Referral follow-up visits	October 2015 – September 2016
Secondary endpoints:		
Appropriate management of malaria, diarrhoea and pneumonia	Treatment record form	October 2015 – September 2016
Factors related to referral	Focus group discussions, KIs Household survey	October 2015 – September 2016

Secondary outcomes:

- Appropriate case management for malaria, pneumonia and diarrhea among children in the private sector.
- Factors which influence the referral or non-referral of sick children from the private sector.

### Sample size estimation

The study will recruit 319 sick children per cluster and the total number of sick children to be recruited from all clusters will be 8910; adjusting for a 10 % loss to follow up and possible withdrawal of private outlets. This was based on assumption that the intervention will increase the primary outcome from 28 to 40 %, at a study power of 80 %, 5 % level of significance and a cluster correlation k, of 0.15; 20 clusters will be required. Ten (10) of them will be randomly allocated to the intervention and 10 to the control arm.

### Study status

The intervention will last one year and 4 months have elapsed since we implemented.

## Discussion

Critical barriers in the implementation of child survival interventions are poor quality of care in the private sector and timely referral and uptake of referral advice at community level. Poor referral of sick children, particularly those that seek care from the private sector is one of the contributory factors to Uganda’s high under-five mortality. The proposed intervention aims to improve referral and uptake of referral advice for children that seek care from private facilities. The barriers to referral may be attributed to inadequate training of providers in the private sector (in diagnosis and management of childhood illnesses); inadequate supervision and regulation; poor linkages and collaboration between the public and private sectors; and non-existent linkages between community structures and the private sector. These barriers will be addressed through an intervention with three components; i) VHTs to raise community awareness on the importance of referral and encouraging families to save money for referral, ii) training and supervision of providers in the private sector to diagnose, treat and refer sick children, iii) regular meetings between the public and private providers to discuss the referral system.

Studies in Uganda and elsewhere have shown that drug shop attendants can be trained to offer appropriate treatement for fever, cough or diarrhea, can procure and store ACTs and RDTs and dispose them off safely [19]. What is currently lacking is the evidence on how to link the private and public sectors. This study will add to ongoing research to demonstrate that such collaboration to improve referral of sick children may be succesful. The immediate impact will be appropriate treatment of sick children. The linkage between the private and public sectors will create a potential avenue for delivery of other public health interventions and improved working relations in the two sectors. Further, improved quality of services in the private sector will improve provider confidence and hopefully more clientelle to the private practices. The long term effect will be reduction in child mortality.

## Conclusions

The data generated from this study will contribute to an understanding of factors of importance for strengthening the referral system, including optimal training required, supervision activities, and community participation. The lessons learned are likely to inform programming at a national and district level to improve referral of children from the private sector.

## Abbreviations

ACTs: Artemensin contain treatment/drugs
iCCM: Integrated community case management
RDTs: Rapid diagnostic test
VHTs: Village heath teams
